# Alterations in step frequency and muscle activities using body weight support influence the ventilatory response to sinusoidal walking in humans

**DOI:** 10.1038/s41598-023-42811-w

**Published:** 2023-09-19

**Authors:** Mako Fujita, Kiyotaka Kamibayashi, Masahiro Horiuchi, Naoyuki Ebine, Yoshiyuki Fukuoka

**Affiliations:** 1 Faculty of Health and Sports Science, Doshisha University, 1-3 Tatara Miyakodani, Kyotanabe, Kyoto 610-0394 Japan; 2 National Institute of Fitness and Sports in Kanoya, Kagoshima, Japan

**Keywords:** Physiology, Environmental sciences

## Abstract

The use of body weight support (BWS) can reveal important insights into the relationship between lower-limb muscle activities and the ventilatory response during sinusoidal walking. Here, healthy participants (n = 15) walked on a treadmill while 0%, 30%, and 50% of their body weight was supported with BWS. The walking speed was varied sinusoidally between 3 and 6 km h^−1^, and three different frequencies, and periods ranging from 2 to 10 min were used. Breath-by-breath ventilation (${\dot{\text{V}}}_{{\text{E}}}$) and CO_2_ output (${\dot{\text{V}}}\text{CO}_{{2}}$) were measured. The tibialis anterior (TA) muscle activity was measured by electromyography throughout the walking. The amplitude (*Amp*), normalized *Amp* [*Amp* ratio (%)], and phase shift (*PS*) of the sinusoidal variations in measurement variables were calculated using a Fourier analysis. The results revealed that the *Amp* ratio in ${\dot{\text{V}}}_{{\text{E}}}$ increased with the increase in BWS. A steeper slope of the ${\dot{\text{V}}}_{{\text{E}}}$–${\dot{\text{V}}}\text{CO}_{{2}}$ relationship and greater ${\dot{\text{V}}}_{{\text{E}}}$/${\dot{\text{V}}}\text{CO}_{{2}}$ values were observed under reduced body weight conditions. The *Amp* ratio in TA muscle was significantly positively associated with the *Amp* ratio in the ${\dot{\text{V}}}_{{\text{E}}}$ (p < 0.001). These findings indicate that the greater amplitude in the TA muscle under BWS may have been a potent stimulus for the greater response of ventilation during sinusoidal walking.

## Introduction

The human body’s respiratory response to exercise is expected to be integrated by several mechanisms, including respiratory feedback from both central and peripheral chemoreceptors^1,2^, central feedforward command^3–5^, and afferent feedback from exercising limb muscles^6–11^. The contribution of feedback from muscle afferents to the ventilatory response during exercise has recently been investigated in humans by using sinusoidal work forcing. For example, a sinusoidal variation of limb-movement frequency by alterations in treadmill speed^12^ or cycling cadence^13^ resulted in a faster phase response and a greater amplitude of the increase in ventilation compared to sinusoidal changes in the treadmill grade or cycling workload. A greater amplitude of the increase in ventilation was also observed when the participants’ stride length was fixed and the step frequency was varied widely in coordination with the sinusoidal changes in treadmill speed^14^. In another investigation, the pedaling cadence variations were closely linked with the respiratory frequency during sinusoidal cycling^15^. These findings support the possibility of a contribution of sinusoidally varying limb movement and frequency (i.e., cadence) to the greater ventilatory responses, perhaps involving upward-moving information from the afferent neurons in exercising muscles^12–14^.

Several studies have used sinusoidal exercise protocols to noninvasively examine factors affecting the ventilatory response in humans^12–14,16–18^. Compared to a constant workload (i.e., a steady-state protocol), a sinusoidal exercise protocol elicits a continuously non-steady-state response so that the kinetic responses of ventilatory and gas exchange variables can be clearly estimated by the phase shift (*PS*) as the time lag and the amplitude (*Amp*) as the magnitude of response^16–18^. Sinusoidal protocols are also considered suitable methods to examine the presence of neural drives at not only exercise onset but also during exercise, though some experimental manipulations are still required in order to partition the central command and afferent feedback components^19^. The present study utilized sinusoidal exercise protocols to specifically determine (i) the kinetic response of lower-limb muscle activities, which may affect the discharge frequency in muscle afferents and the consequent ventilatory response after muscle contractions^2,7,10^, and (ii) the link between the muscle activities and ventilation during sinusoidal walking.

One approach that can be applied to manipulate muscle activities and examine the relationship between muscle activities and ventilatory response during sinusoidal walking is to perform experiments using a body weight support (BWS) system. BWS can provide a simulation of reduced gravity and/or reduced body weight during walking^20–22^ and running^23–25^. The effects of reduced gravity on walking mechanics and energetics have been examined. For example, several research groups that used a BWS system with a torso harness observed a decrease in the activity of ankle extensors (e.g., gastrocnemius and soleus muscles) when the body weight support was increased, whereas the activity of the dorsi-flexor (tibialis anterior [TA]) muscle in the swing phase showed only a slight decrease or no change with an increase in body-weight support^22,26,27^. These observations demonstrated that the load sensitivity is more predominant in extensors than in dorsi-flexors^26,28^.

It has also been shown that the decrease in gravity at a given walking speed caused a small reduction in the stride length, which occurred almost exclusively because of a decrease in the stance time rather than the swing time^29^. This indicates that the swing time is not determined by a contribution of gravity^29^. The effects of BWS on locomotion parameters such as the step frequency and stride length in healthy humans have also been described^30–32^, but it remains unclear whether the increase or decrease in lower-limb muscle activities and such locomotion parameters under BWS conditions affect the ventilatory response during walking. We speculated that when combined with sinusoidal exercise protocols, BWS may provide a clear indication of the linkage between the muscle activities (i.e., neural drives from working muscle) and the ventilatory response during sinusoidal walking.

We thus conducted the present study to examine the relationship between the lower-limb muscle activities and ventilation in response to sinusoidal walking under simulated reduced body weight conditions in healthy male participants. It is considered that there is a minimal possibility that sex differences exist in the ventilatory response in our present experimental protocol. This assumption was grounded in evidence that the sex difference has not been detected in both the ventilatory response to sinusoidal protocols^12,13^ and the muscle activity under body weight support^20^. We hypothesized that lower-limb muscle activities are associated with the ventilatory response during sinusoidal walking, possibly involving afferent feedback from exercising limb muscles^10,33–35^. To test this hypothesis, we manipulated the participants’ muscle activities by applying three different BWS conditions. We expected that an increase in BWS may decrease the activity of anti-gravity muscles such as the gastrocnemius and soleus muscles^22,26,27^ but increase the magnitude of the response in the relative TA muscle activity. This may be related to the increase in step frequency as a consequence of the reduction in stride length under reduced body weight conditions^29^. We also speculated that the greater relative TA muscle activity under reduced body weight conditions may be associated with a greater ventilatory response during sinusoidal walking, perhaps involving the muscle afferent activation within the TA muscle. To test these hypotheses, we compared the lower-limb muscle activities and ventilatory and gas exchange variables in response to sinusoidal changes in walking speed among several BWS conditions.

## Participants and methods

### Participants

The participants were 15 healthy young male volunteers (age 20 ± 1 years, height 173 ± 5 cm, weight 65.2 ± 4.6 kg, mean ± standard error [SE]), who were not taking any medication and had no prior history of cardiometabolic disease that could affect cardiorespiratory responses. They were also non-smokers and had not been engaging in regular exercise. They were fully informed of any potential risks and discomfort associated with the experiments before they provided their written informed consent to participate in the study. Consent was obtained for publication of identifiable images of research participants. All methods in this study were approved by the Ethics Committee of the Institutional Review Board of Doshisha University (no. 19033) and were conducted in accord with the Declaration of Helsinki.

### Experimental overview

Each participant visited the laboratory on six occasions over a maximum timespan of 5 weeks. The participants performed two sinusoid sessions under the three body weight support (BWS) conditions described below. The order of the three BWS conditions was randomized, and the participants performed one of these experimental measurements on one experimental day [total of six visits (two sinusoid sessions × three BWS conditions)]. The experimental protocol consisted of sinusoidal variations in treadmill speed while the participant’s body weight was lifted by the BWS system. Cardiorespiratory, locomotive, and electromyography (EMG) variables were measured throughout the protocols as described below. The study was designed to obtain repeated within-subject measurements.

### The body weight support (BWS) system

**Figure 1:**
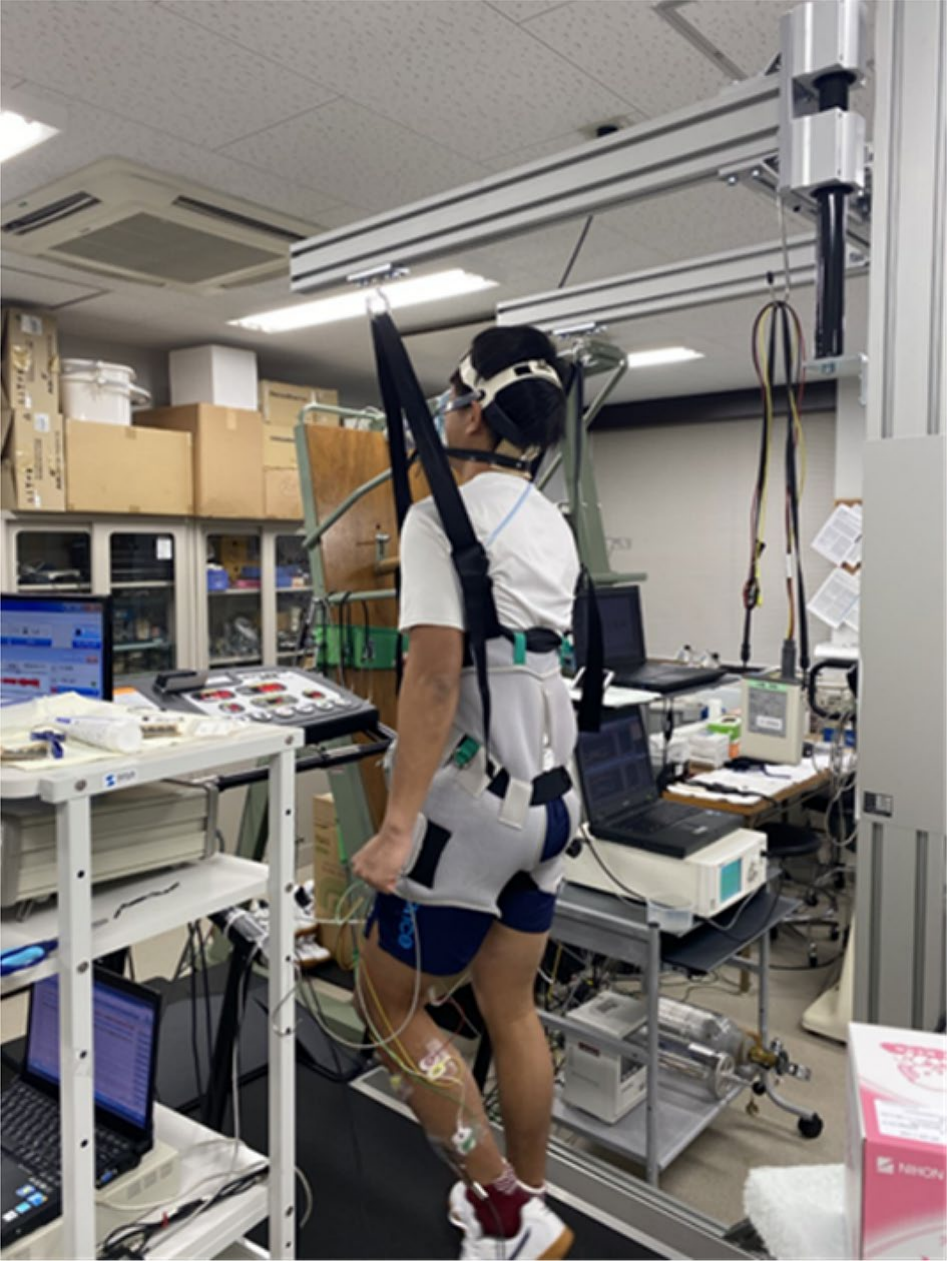
The custom-made body weight support (BWS) apparatus. The participant’s body weight was reduced by 0%, 30%, and 50% without disturbing the normal leg-swing motion during walking.

A custom-made body suspension apparatus lifted the participant’s torso by an elastic rehabilitation harness (Fig. 1) that was installed around a motor-driven treadmill (Well Road 200E; Takei Scientific Instruments, Niigata, Japan). The same apparatus was used in our earlier studies^21,25^. The participant’s body weight (BW) was reduced by 0% (100% of his BW remained; 100%BW), 30% (70% of his BW: 70%BW), and 50% (50% of his BW: 50%BW) based on previous studies^20,21,25,26^. A load cell (TSA-110; Takei Scientific Instruments) was positioned between a control box and a spring (with 30 cm free length and a spring constant of 5.7 kg cm^−1^) to measure the actual delivered force. The participant’s BW was lifted by the spring system before he began the walking protocols, and he walked while wearing the harness even under the 100%BW condition (in which none of his BW was lifted). This BWS system allowed normal leg swing during walking^21,25^.

### Experimental protocol

**Figure 2:**
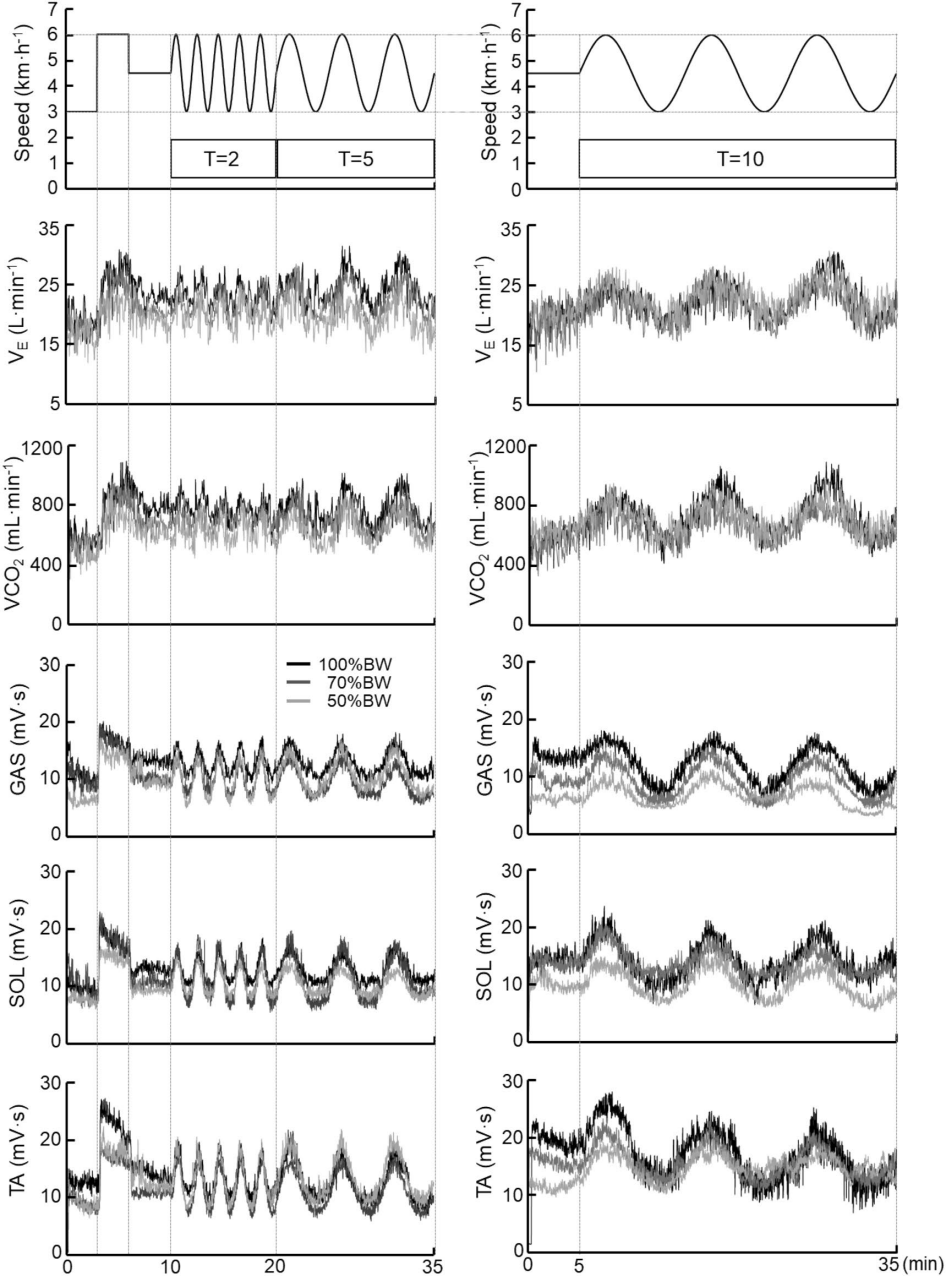
Sinusoidal walking protocols at a sinusoidal speed between 3 and 6 km min^−1^ at the periods of 2 and 5 min (left panel) and 10 min (right panel). The interpolated 1-s data of pulmonary ventilation (${\dot{\text{V}}}_{{\text{E}}}$), the CO_2_ output (${\dot{\text{V}}}\text{CO}_{{2}}$), and the muscle activities in the time series at each walking protocol are shown for all three BWS conditions (100%BW, 70%BW, and 50%BW) in a representative participant. *GAS* gastrocnemius, *SOL* soleus, *TA* tibial anterior.

All experimental tests were completed in a temperature-controlled laboratory (25 ± 0.4 °C with 50 ± 3% relative humidity), and all participants wore underwear, shorts, and a T-shirt, as well as socks and shoes. The sinusoidal walking protocols used herein are based on our earlier work^14,18^. The treadmill speed was varied in a sinusoidal pattern from 3 to 6 km h^−1^ with three different frequencies, the periods (*T*) of 2, 5, and 10 min (Fig. 2). At the beginning of the sinusoidal walking, a stepwise manner (steady-state) constant workload at speeds of 3, 6, and 4.5 km h^−1^ was carried out for a total of 10 min. The purposes of this manipulation were to (i) improve the participants’ adaptation to the workload and (ii) measure the magnitude of the increases in cardiorespiratory variables^18,36^.

The sinusoidal loading at 2-min periods was repeated for five cycles (2 × 5 = 10 min) followed by 5-min periods for three cycles (5 × 3 = 15 min) (Fig. 2). On another day, another sinusoidal loading at 10-min periods was repeated for three cycles (10 × 3 = 30 min) after a constant workload at the midpoint between the maximum and minimum of 5 min (Fig. 2). Each sinusoidal period of oscillation was studied on a separate occasion (one session at a time and two sessions per week for each participant). The participants walked on the treadmill at a freely chosen pace on the level (± 0%) gradient. A microcomputer transmitted the signal controlling the speed of the motor-driven treadmill through a digital-analog converter.

The participants completed the walking protocols while their BW was supported by the BWS system. The order of the three BWS conditions was randomized, and the participants performed one of these experimental measurements on one experimental day [total of six visits (two sinusoid sessions × three BWS conditions) for each participant].

### Measurements

A mass-flow sensor (type AB, Minato Medical Sciences, Osaka, Japan) was fit to the expiratory port of the valve of the face mask worn by the participant to continuously record the participant’s expiratory airflow, which was calibrated before each measurement with a 3-L syringe at three different flow rates in accord with manufacturer’s instructions. The pulmonary ventilation (${\dot{\text{V}}}_{{\text{E}}}$; L min^−1^), oxygen uptake (${\dot{\text{V}}}\text{O}_{{2}}$; mL min^−1^) (STPD, i.e., standard temperature [0 °C or 273 K] and pressure [760 mmHg] and dry [no water vapor]), carbon dioxide output (${\dot{\text{V}}}\text{CO}_{{2}}$; mL min^−1^) (STPD), tidal volume (VT; mL), breathing frequency (B*f*; breaths min^−1^), and end-tidal carbon dioxide pressure (P_ET_CO_2_; mmHg) were determined using a computerized online breath-by-breath measuring system (AE310S, Minato Medical Sciences). The second-by-second time course was calculated for each variable by interpolation of the breath-by-breath data. Two sets of reference gases of known concentrations (O_2_: 15.04%, CO_2_: 2.92%, and N_2_: 82.04%; O_2_: 11.93%, CO_2_: 6.96%, and N_2_: 81.11%) and room air were used to calibrate the gas analyzer.

An electrocardiogram (ECG) was recorded using a bio-amplifier (BA1104m, Nihon Santeku Co., Osaka). The R-R intervals during sinusoidal work were calculated beat-by-beat by the computer, and 1-s interval heart rate (HR) data were measured from the calculated R-R intervals (R-R) and converted as HR values (60/R-R).

The step frequency and stride length of each participant were measured in each protocol with the use of a pre-built and positionally standardized sensor that was enclosed by an activation switch on the sole of the right foot^18^. We defined the stride length as the distance from the right initial contact (heel strike) to the consecutive right initial contact. We calculated the stride length knowing the treadmill speed and step frequency. The signal from the stepping sensor was fed into a data acquisition system (PowerLab system, A/D Instruments, Castle Hill, NSW, Australia) and temporally aligned with the ventilatory and ECG data. The step frequency and stride length data were interpolated into a 1-s interval value before the Fourier analysis.

For the EMG measurements, pre-amplified active surface EMG electrodes (BA-U410m; Nihon Santeku Co.) were placed on the tibialis anterior (TA), the lateral head of the gastrocnemius (GAS), and the soleus (SOL) muscles^25^. Before the electrode placement, the skin was shaved with the use of abrasive gel (skinPure, YZ-0019; Nihon Kohden, Tokyo) and wiped with alcohol for exfoliation. We placed the sensors on the belly of the appropriate muscles (except for the SOL muscle, in which the sensor was placed below the belly of the muscle to minimize crosstalk with the GAS muscle). The electrodes were secured using surgical tape to avoid disturbing the locomotor tasks.

The EMG signals were amplified with a bio-amplifier (BA 1104 m; Nihon Santeku Co.). The sampling frequency was set at 2 kHz, and a band-pass filter (8–500 Hz) was applied for the EMG signals. All signals from the EMG were simultaneously recorded throughout the whole protocol and fed into the PowerLab data acquisition system. The recorded EMG signals were integrated (iEMG) over 0.5-s intervals and were considered the measure of muscle activity throughout the sinusoidal walking.

### Data management

All of the cardiorespiratory data, locomotive data (step frequency and stride length), and iEMG data were analyzed using a standard Fourier analysis as described^14,18,35^. The breath-by-breath ventilatory and gas exchange data were interpolated into a second-by-second interval value using a linear interpolation method supported by graphing and data analysis software (KaleidaGraph ver. 4; Synergy Software, Reading, PA, USA) before the Fourier analysis. The qualities of data and outliers were checked using the same software. The repeated sinusoidal cycles were superimposed and averaged. The variation in the speed of the treadmill was regarded as the input function. The amplitude (*Amp*) and the phase shift (*PS*; degree) of the fundamental component (the same frequency as the input function) of the cardiorespiratory responses, locomotive responses, and the iEMG responses were computed as follows:

1 $$Amp =\sqrt{{Re}^{2}+{Im}^{2}}$$

and

2 $$PS = \tan^{ - 1} \left( {\frac{Re}{{Im}}} \right)$$

The *Amp* represents the magnitude of the increase or decrease in the physiological variable from the mean (i.e., the midpoint of the sine wave); the larger the *Amp*, the higher the response. The *PS* refers to the time delay behind a sinusoidal stimulus; the larger the *PS*, the slower the response. The *Re* and *Im* are the real and imaginary parts of these responses determined after a second-by-second interpolation of these responses (*x*) as:

3 $$Re=\frac{2}{NT}\sum \limits_{t=0}^{NT}\left[\left(x\left(t\right)-Mx)\mathrm{ cos}(2\uppi f\mathrm{t}\right)\right]$$

and

4 $$Im=\frac{2}{NT}\sum\limits _{t=0}^{NT}\left[\left(x(t)-Mx)\mathrm{ sin}(2\uppi f\mathrm{t}\right)\right]$$

where *x*(*t*) is the response value at time *t* (in s), and *Mx* is the mean value of *x* for an integer number of cycles (*N*) or the point at which the sine wave intersects the y-axis (i.e., the midpoint of the sine wave). *T* is the period of the input signal (in s), and *f* (= 1/*T*) is its frequency in cycles per second.

We normalized the *Amp* responses of sinusoidal variations in the cardiorespiratory, locomotive, and iEMG variables to correct for expected differentials between individuals. The *Amp* values were divided by the magnitude of each parameter obtained during steady-state walking from 3 to 6 km h^−1^. The differences in the average data of the final 1 min of each period of steady-state walking at 3 and 6 km h^−1^ were regarded as the magnitude. The normalized *Amp* is presented as the *Amp* ratio (%)^18^.

### Statistical analyses

We performed a two-way repeated measures analysis of variance (ANOVA) to test for significant differences in each cardiorespiratory and locomotive (step frequency and stride length) variable. The factors were the BWS conditions (50%, 70%, and 100%BW) and the frequency period (*T*; 2, 5, and 10 min). A one-way ANOVA with the BWS conditions as a factor was used to assess differences in the averaged *Mx*, *Amp*, and *PS* values regardless of the frequency periods in cardiorespiratory variables and in muscle activities. Bonferroni’s test was applied for the appropriate datasets if a significant F-value was obtained. Pearson’s correlation coefficient was obtained to estimate the relationship between the *Amp* ratio in ${\dot{\text{V}}}\text{CO}_{{2}}$ and ${\dot{\text{V}}}_{{\text{E}}}$, and between the *Amp* ratio in each muscle and ${\dot{\text{V}}}_{{\text{E}}}$. The results are presented as the mean ± standard error (SE). Statistical significance was set at p < 0.05.

We used a sample size determination program (PS: Power and Sample Size Calculation ver. 3.1.6 by D.D. William and D.P. Walton) to determine whether a sample size is adequate to detect differences among BWS conditions. The present study was designed to analyze a continuous response variable from matched pairs of participants. In our previous study^25^, the difference in the TA muscle response of matched pairs within two conditions (control and 30% body weight unloading) was normally distributed with standard deviation of 2.65. If the true difference in the mean response of matched pairs is 2.5, it is necessary to study at least 11 pairs of subjects to be able to reject the null hypothesis that this response difference is zero with the probability (power) value 0.8. The probability of Type I error associated with this test of the null hypothesis is 0.05.

### Ethics statement

This study was reviewed and approved by the Ethic Committees of the Institutional Review Board of Doshisha University (no. 19033). The participants provided their written informed consent to participate in this study.

## Results

### Locomotion variables

**Figure 3:**
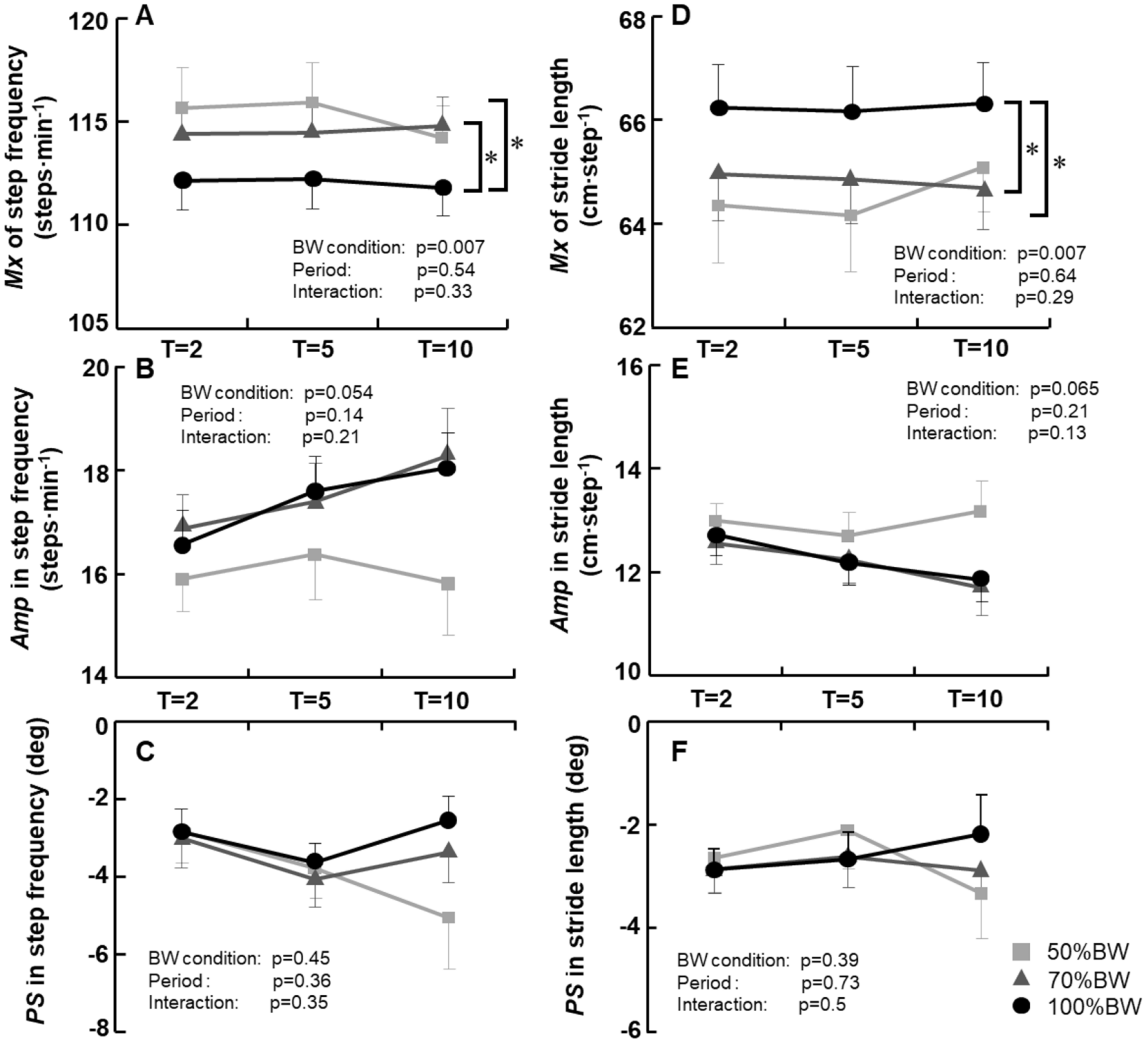
The mean (*Mx*), amplitude (*Amp*), and phase shift (*PS*) values in the step frequency (**A**–**C**) and stride length (**D**–**F**) under the three BWS conditions. T2, 5, and 10 indicate sinusoidal frequency periods of 2, 5, and 10 min, respectively. ^†^p < 0.05 50%BW vs. 100%BW. ^#^p < 0.05 70%BW vs. 100%BW. Data are mean ± standard error (SE).

The *Mx*, *Amp*, and *PS* values of the participants’ step frequency and stride length during sinusoidal walking under the three BWS conditions (50%, 70%, and 100% body weight) at all periods (T: 2, 5, and 10 min) are depicted in Fig. 3. Compared to the 100%BW condition, the *Mx* values of the step frequency were significantly greater under the 50%BW and 70%BW conditions (BW condition effect: p = 0.007), whereas no interaction effect was revealed (Fig. 3A). In contrast, the *Mx* of the stride length under the 50%BW and 70%BW conditions were significantly lower than that under the 100%BW condition (BW condition effect: p = 0.007) without an interaction effect (Fig. 3D).

No significant main effects for the BW condition and period, or interaction effects were observed in the *Amp* and *PS* in the step frequency and stride length (Fig. 3B,C,E,F).

### Mean values of cardiorespiratory variables

The averaged *Mx* values regardless of the frequency period in cardiorespiratory variables are given in Table 1. The averaged *Mx* of ${\dot{\text{V}}}\text{O}_{{2}}$ under the 70%BW and 50%BW conditions was significantly decreased compared to the 100%BW condition (F = 42.382, p < 0.001). The averaged *Mx* of ${\dot{\text{V}}}\text{CO}_{{2}}$ was also significantly decreased under the 70%BW and 50%BW conditions compared to the 100%BW condition (F = 32.393, p < 0.001). The averaged *Mx* of ${\dot{\text{V}}}_{{\text{E}}}$ decreased in proportion to the increase in BWS, along with a significant difference between the 50%BW and 100%BW conditions (F = 6.024, p = 0.004).

**Table 1:** The averaged *Mx* values regardless of the period in of cardiorespiratory variables during sinusoidal walking under three body weight support conditions.

BWS	${\dot{\text{V}}}\text{O}_{{2}}$(mL min^−1^)	${\dot{\text{V}}}\text{CO}_{{2}}$(mL min^−1^)	HR (beats min^−1^)	${\dot{\text{V}}}_{{\text{E}}}$(L min^−1^)	${\dot{\text{V}}}_{{\text{E}}}$/${\dot{\text{V}}}\text{CO}_{{2}}$	P_ET_CO_2_ (mmHg)	VT (L)	B*f* (breaths min^−1^)
100%BW	882 ± 12^†#^	800 ± 13^†#^	98.0 ± 14.0^†#^	24.5 ± 0.6^†^	30.4 ± 0.5	42.9 ± 0.4	1.04 ± 0.04^†#^	24.8 ± 1.0
70%BW	823 ± 13	751 ± 13*	92.7 ± 12.7	24.0 ± 0.6	31.9 ± 0.6^#^	42.3 ± 0.4	0.97 ± 0.03*	25.6 ± 1.1
50%BW	802 ± 13	717 ± 13	91.1 ± 12.0	23.2 ± 0.5	32.3 ± 0.6^†^	41.8 ± 0.4	0.92 ± 0.03	26.2 ± 1.0^†^

*p < 0.05 50%BW vs. 70%BW.

^†^p < 0.05 50%BW vs. 100%BW.

^#^p < 0.05 70%BW vs. 100%BW.

Data are mean ± standard error (SE).

*Bf* breathing frequency, *BWS* body weight support, *HR* heart rate, *Mx* mean values of x, *P*_*ET*_*CO*_*2*_ partial pressure of end-tidal CO_2_, ${\dot{\text{V}}}\text{CO}_{{2}}$ carbon dioxide output, ${\dot{\text{V}}}_{{\text{E}}}$ pulmonary ventilation, ${\dot{\text{V}}}\text{O}_{{2}}$ oxygen uptake, *VT* tidal volume.

In addition, the ${\dot{\text{V}}}_{{\text{E}}}$/${\dot{\text{V}}}\text{CO}_{{2}}$ values were significantly greater under the 50%BW and 70%BW conditions compared to that under the 100%BW condition (F = 14.691, p < 0.001). No differences in P_ET_CO_2_ across the conditions were observed.

### Ventilatory variables

**Figure 4:**
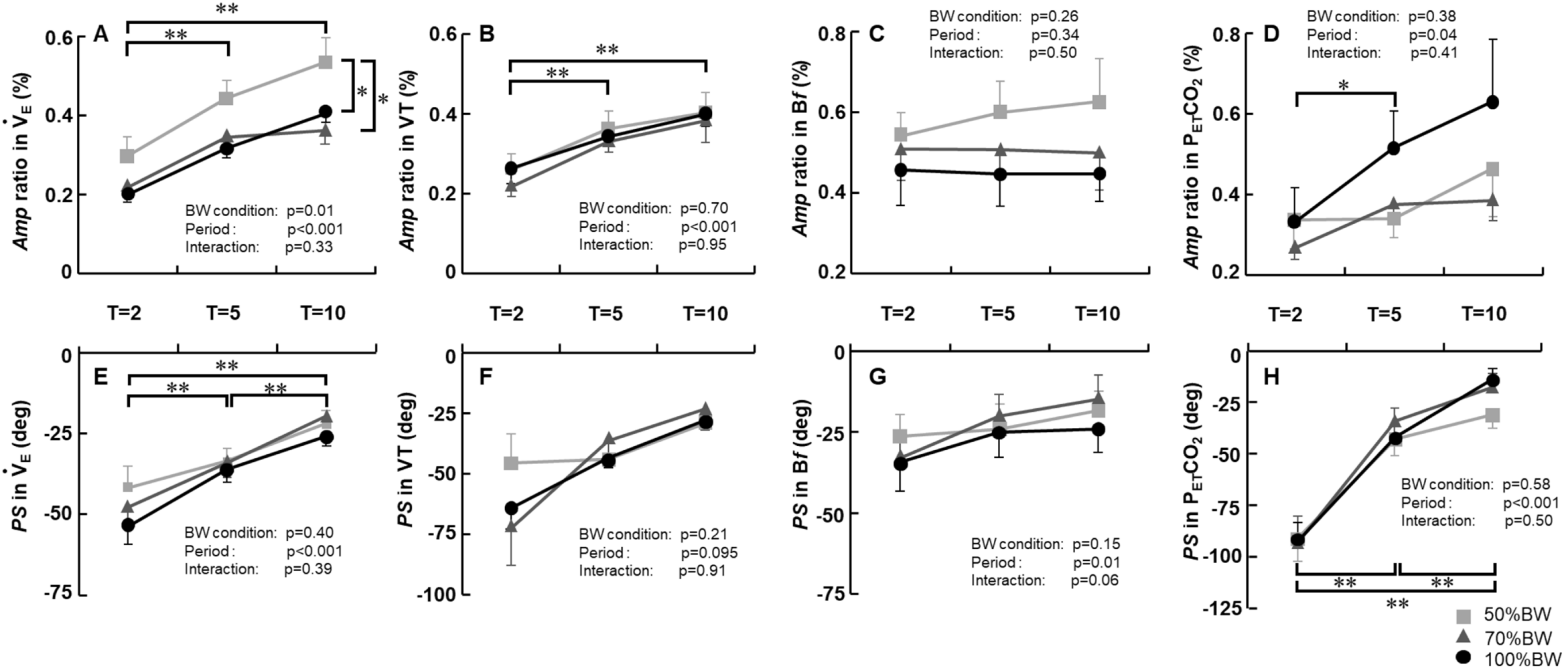
Comparisons of the *Amp* ratio and the *PS* in ${\dot{\text{V}}}_{{\text{E}}}$, tidal volume (VT), breathing frequency (B*f*), and the partial pressure of end-tidal CO_2_ output (P_ET_CO_2_) under the three BWS conditions. *p < 0.05 50%BW vs. 70%BW. ^†^p < 0.05 50%BW vs. 100%BW. Data are mean ± SE.

Figure 4 presents the *Amp* ratio [i.e., the normalized *Amp* (%)] and the *PS* response of ventilatory variables to the sinusoidal workload at each period under the three BWS conditions. There were significant main effects of the BW condition and the period on the *Amp* ratio in ${\dot{\text{V}}}_{{\text{E}}}$ (BW condition effect: p = 0.01, period effect: p < 0.001). In addition, the *Amp* ratio in ${\dot{\text{V}}}_{{\text{E}}}$ under the 50%BW condition was significantly greater compared to those under the 70%BW and 100%BW conditions (Fig. 4A). The *Amp* ratio in VT and in P_ET_CO_2_ significantly changed with the period (p < 0.001 and p < 0.05, respectively) but with no main effect of the BW condition and with no interaction effect (all p > 0.05, Fig. 4B,D). The *Amp* ratio of B*f* was slightly increased under the 50%BW condition, whereas no significant effects of the BW condition and the period were detected (Fig. 4C).

We did not observe any effects of the BWS condition on the *PS* response in ${\dot{\text{V}}}_{{\text{E}}}$, VT, B*f*, or P_ET_CO_2_, but there were period effects on the PS response in ${\dot{\text{V}}}_{{\text{E}}}$, B*f*, and P_ET_CO_2_ (Fig. 4E–H).

### Integrated EMG variables

Table 2 provides the averaged *Mx*, *Amp*, and *PS* values regardless of periods in each muscle’s activities. When the data from all of the periods were averaged, the *Mx* values of the GAS muscle activity under the 50%BW and 70%BW conditions were significantly decreased compared to those under the 100%BW condition (F = 8.508, p < 0.001). The *Mx* values of the SOL and TA muscle activity were not affected by BWS.

**Table 2:** The averaged *Mx*, *Amp* ratio, and *PS* values regardless of the period in lower limb muscles during sinusoidal walking under the three BWS conditions.

	GAS	SOL	TA
*Mx* (mV s)			
100%BW	12.54 ± 1.08^#†^	17.00 ± 1.34	15.27 ± 1.03
70%BW	8.26 ± 0.89	15.16 ± 1.71	15.01 ± 1.42
50%BW	7.14 ± 0.82	13.46 ± 1.04	13.58 ± 1.08
*Amp* ratio (%)			
100%BW	0.55 ± 0.09	0.48 ± 0.03	0.50 ± 0.05
70%BW	0.49 ± 0.04	0.70 ± 0.11*	0.57 ± 0.09
50%BW	0.41 ± 0.02	0.45 ± 0.02	0.77 ± 0.15
*PS* (deg)			
100%BW	-3.5 ± 5.3	-6.9 ± 6.0	-7.6 ± 5.2
70%BW	2.9 ± 3.0	8.3 ± 4.2	3.2 ± 4.2
50%BW	3.9 ± 2.1	2.7 ± 3.2	4.4 ± 2.8

Data are mean ± SE.

*Amp* amplitude, *GAS* gastrocnemius, *PS* phase shift, *SOL* soleus, *TA* tibial anterior.

*p < 0.05 50%BW vs. 70%BW.

^†^p < 0.05 50%BW vs. 100%BW.

^#^p < 0.05 70%BW vs. 100%BW.

The *Amp* ratio in the TA muscle increased with the increase in the BWS, while the *Amp* ratio in the GAS muscle decreased with the increase in BWS; however, these changes in the *Amp* ratio were not significant for either of the muscles (Table 2).

The *PS* for the muscle activities tended to be smaller in the 50%BW and 70%BW conditions compared to the 100%BW condition, but not significantly in any of the three muscles.

### The relationship between the *Amp* ratios in ${\dot{\text{V}}}_{{\text{E}}}$ and ${\dot{\text{V}}}\text{CO}_{{2}}$ and the muscle activities

**Figure 5:**
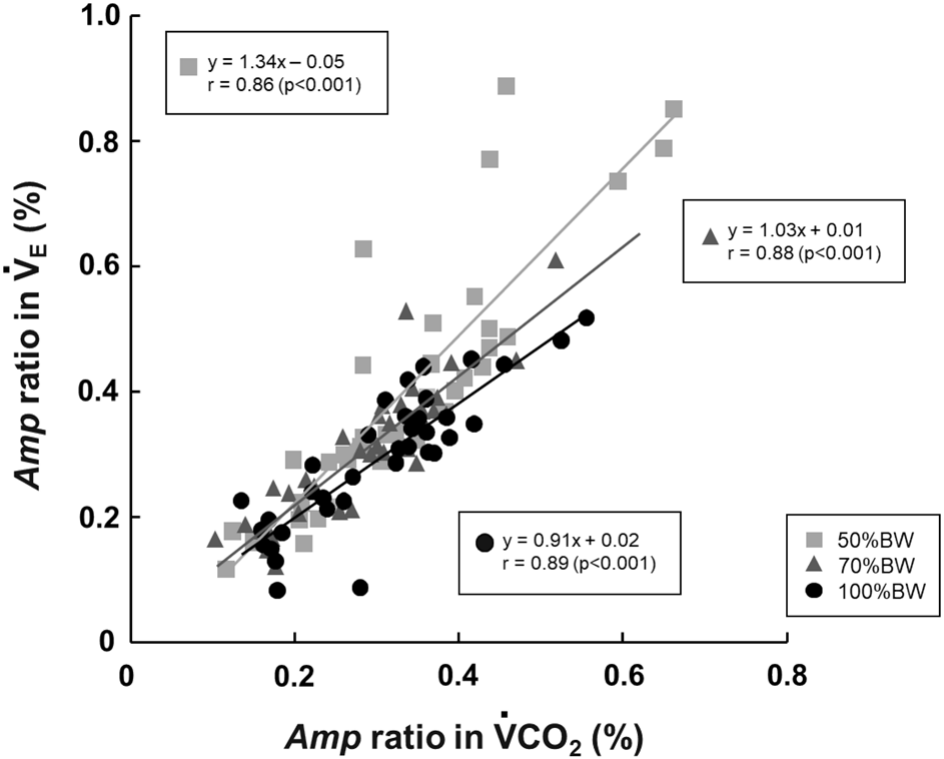
The relationship between the *Amp* ratio in ${\dot{\text{V}}}_{{\text{E}}}$ and ${\dot{\text{V}}}\text{CO}_{{2}}$ under the 100%BW, 70%BW, and 50%BW conditions, respectively when the data of all periods are pooled.

**Figure 6:**
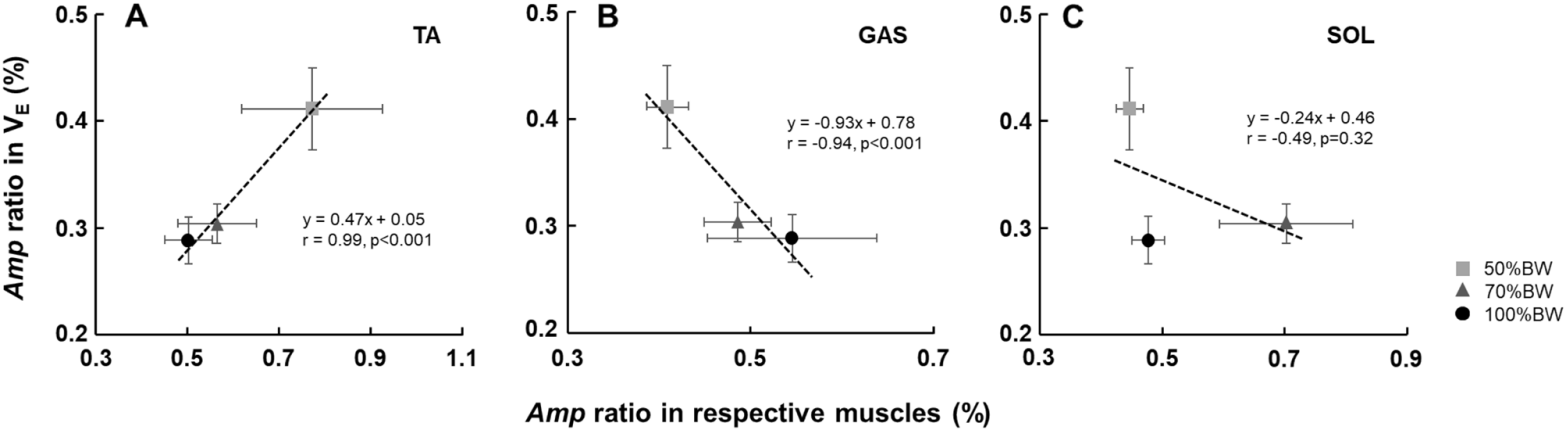
The relationship between the averaged *Amp* ratio regardless of the period in ${\dot{\text{V}}}_{{\text{E}}}$ and the lower-limb muscle activities.

The *Amp* ratio in the ${\dot{\text{V}}}_{{\text{E}}}$ was closely related to the *Amp* ratio in the ${\dot{\text{V}}}\text{CO}_{{2}}$ when the data from all periods were pooled (50%BW: r = 0.86, 70%BW: r = 0.88, and 100%BW: r = 0.89, p < 0.001) (Fig. 5). The slope of the regression lines of the ${\dot{\text{V}}}_{{\text{E}}}$–${\dot{\text{V}}}\text{CO}_{{2}}$ relationship under the 50%BW condition was the steepest (slope [s]: 1.34), followed by the 70%BW condition (s: 1.03) and the 100%BW condition (s: 0.91). Moreover, the averaged *Amp* ratio regardless of the periods in ${\dot{\text{V}}}_{{\text{E}}}$ showed a significant positive correlation with the averaged *Amp* ratio in the TA muscle activity (r = 0.99, p < 0.001) (Fig. 6A). There was also a negative correlation between the averaged *Amp* ratio in ${\dot{\text{V}}}_{{\text{E}}}$ and the GAS muscle activity (r = − 0.94, p < 0.001) (Fig. 6B). No significant correlation was detected between the averaged *Amp* ratio in ${\dot{\text{V}}}_{{\text{E}}}$ and SOL activity (Fig. 6C).

## Discussion

The primary findings of this study are three-fold: (i) the *Amp* ratio in ${\dot{\text{V}}}_{{\text{E}}}$ was significantly greater under the 50%BW condition compared to the other two BWS conditions, (ii) the slope of the *Amp* ratio of the ${\dot{\text{V}}}_{{\text{E}}}$ and ${\dot{\text{V}}}\text{CO}_{{2}}$ relationship was steeper under the 50%BW condition compared to the other two BWS conditions despite the similar P_ET_CO_2_ values, and (iii) the averaged *Amp* ratio regardless of the period in ${\dot{\text{V}}}_{{\text{E}}}$ was positively associated with the averaged *Amp* ratio in the TA muscle activity. These results support our hypothesis that the TA muscle responded to a greater extent with the increase in BWS, whereas the other two muscles decreased their activities with the BWS. We speculated that the TA muscle may have activated during our participants’ sinusoidal walking due to the significant increase in step frequency under BWS, whereas BWS attenuated the activities of the other two muscles, which are the anti-gravity muscles.

We had also hypothesized that the greater relative TA muscle activity under BWS may be associated with a greater ventilatory response. Our observations of the greater ventilatory outcomes (the *Amp* ratio in ${\dot{\text{V}}}_{{\text{E}}}$ and the ${\dot{\text{V}}}_{{\text{E}}}$–${\dot{\text{V}}}\text{CO}_{{2}}$ relationship) and the positive linear relationship between the amplitudes of ${\dot{\text{V}}}_{{\text{E}}}$ and the TA muscle suggest that the ventilatory response to sinusoidal changes in treadmill speed is driven by the greater TA muscle response under BWS conditions.

### BWS and the mean value of ventilation during sinusoidal walking

We observed a reduction in the *Mx* of the metabolic rate (${\dot{\text{V}}}\text{O}_{{2}}$ and ${\dot{\text{V}}}\text{CO}_{{2}}$) with the increase in BWS and ${\dot{\text{V}}}_{{\text{E}}}$ under the 50%BW condition (Table 1). Several research groups have examined the metabolic cost of walking under simulated reduced-gravity conditions by using torso suspension with an elastic harness^21,23,37,38^ or lower-body positive pressure^39^, and they reported that the metabolic rate during walking decreases at lower gravity values^21,23,37–39^. Our present observation of statistically significant decreases in the *Mx* values of ${\dot{\text{V}}}\text{O}_{{2}}$ and ${\dot{\text{V}}}\text{CO}_{{2}}$ under the 70%BW and 50%BW conditions compared to the 100%BW condition was consistent with the results of previous studies^21,23,37,38^. However, the small separation in absolute *Mx* values should be considered within the context of the variability in measurements of the metabolic rate, which could be influenced by biological variation, instrument error, and/or pure error such as human mistakes^40–42^.

On the other hand, our observation of a relatively small (but statistically significant) percentage reduction in the metabolic cost despite the participants’ body weight being decreased by 30% and 50% during walking was in line with the results of prior investigations^23,37,38^. It was predicted that the efficiency of exchanging gravitational potential energy with forward kinetic energy of the center of mass would be decreased during walking at lower gravity^23,43^, and thus the locomotion muscles may have to do more mechanical work to compensate for an ineffective exchange of mechanical energy during walking^23,43^. It is thus conceivable that the metabolic rate during walking under simulated reduced gravity does not decrease in the same manner as the percentage of body weight reduction. It is also conceivable that the reduction in metabolic cost during walking with increasing BWS resulted in the relatively small but statistically significant reduction in the *Mx* of ${\dot{\text{V}}}_{{\text{E}}}$ observed in the present study.

In contrast, we observed a greater ${\dot{\text{V}}}_{{\text{E}}}$/${\dot{\text{V}}}\text{CO}_{{2}}$ value with the increase in BWS while the *Mx* of P_ET_CO_2_ remained unchanged irrespective of the BWS conditions (p = 0.37) (Table 1). In this study, we used P_ET_CO_2_ value to estimate PaCO_2_ to predict the control of ${\dot{\text{V}}}_{{\text{E}}}$ during sinusoidal walking under the three BWS conditions^44,45^. While differences between P_ET_CO_2_ and PaCO_2_ during exercise due to the increase in dead space and the within-breath fluctuations of alveolar gas composition have been discussed^44^, an indirect estimation of PaCO_2_ from P_ET_CO_2_ is often used to predict the CO_2_-mediated ventilatory control in healthy humans^2,45–47^. Therefore, our observation regarding the ${\dot{\text{V}}}_{{\text{E}}}$/${\dot{\text{V}}}\text{CO}_{{2}}$ value and the *Mx* of P_ET_CO_2_ indicates that the control of ${\dot{\text{V}}}_{{\text{E}}}$ under the three BWS conditions appeared to have increased via the chemoreflex to maintain a given level of PaCO_2_ homeostasis^2,47,48^, despite reduced alveolar ventilation that may be due to the preferential increase of breath frequency (B*f*) over tidal volume with the increase in BWS (Table 1).

### BWS and the *Amp* response of ventilation during sinusoidal walking

We also observed that the *Amp* ratio of ${\dot{\text{V}}}_{{\text{E}}}$ was significantly greater under the 50%BW condition compared to the other two BWS conditions, and this may be attributable to significant increases in step frequency with increasing BWS. Several groups have examined the ventilatory response during leg cycling^13,17,19^ or treadmill walking^12^ while the workload was changed by altering either the limb loading (pedal force or treadmill grade) or the speed of exercise (pedaling rate/cadence or treadmill speed), and those groups reported that a fast limb-movement frequency increases the ventilatory response (greater amplitude and lower phase shift for ${\dot{\text{V}}}_{{\text{E}}}$) during exercise. Our recent investigation regarding the influence of step frequency on the ventilatory response showed the greater amplitude of the increases in ventilation and B*f* when the stride length was fixed, and the step frequency was largely varied in coordination with the sinusoidal changes in treadmill speed^14^. Our present findings, demonstrating a greater *Amp* of ${\dot{\text{V}}}_{{\text{E}}}$ under the 50%BW condition compared to the other two BW conditions (Fig. 4A) despite the similar *PS* of ${\dot{\text{V}}}_{{\text{E}}}$ (Fig. 4E) while showing a greater *Mx* of step frequency under the 50%BW and 70%BW conditions compared to the 100%BW (Fig. 3A) are partially consistent with the results from those earlier studies^12–14,17,19^.

In addition, our present finding of the slight, nonsignificant increase in the *Amp* of B*f* under the 50%BW condition is somewhat consistent with reported results^15,49^, suggesting that muscle afferent feedback plays a primary role in regulating B*f* during passive exercise at moderate intensity^15^. The VT and B*f* are presumably regulated by different inputs^50^: the VT seems to be regulated mainly by metabolic inputs, whereas the B*f* appears to be regulated by fast (neural) inputs, including efferent feedforward input (i.e., central command) and group III/IV muscle afferent feedback^9,10,50^.

We thus suspect that the alteration in our participants’ limb movement (i.e., the significant increase in step frequency) under BWS may have affected the ${\dot{\text{V}}}_{{\text{E}}}$ and B*f* responses during sinusoidal walking. Possible mechanisms underlying these observations involve the group III and IV afferent neurons in exercising limbs that are stimulated by muscle contraction^33,34^. The group III and IV afferents are considered ꞌmechanoꞌ and/or ꞌmetaboꞌ receptors that detect muscle contraction and/or changes in muscle metabolites, and they are thought to affect the ventilatory and cardiovascular response to rhythmic exercise in humans^10^. In the present study, the group III and IV afferents in exercising muscles may have activated during sinusoidal walking when the step frequency was increased with the increase in BWS and accelerated the ${\dot{\text{V}}}_{{\text{E}}}$ and B*f* responses to sinusoidal walking.

However, the contribution of efferent feedforward inputs to the ventilatory response should also be considered, as central command is not ruled out in the present study. As a mechanism by which descending motor signals from the higher central nervous system including the hypothalamus activate both locomotor and cardiorespiratory neurons at the onset of exercise^3,10^, central command has been investigated in humans by manipulating the amount of central motor drive required to perform a given physical task^51–53^. The results provided indirect evidence that the magnitude of the central motor drive affects the cardiorespiratory response. The identification of the contribution of central command and muscle afferent feedback to ventilatory regulation is complicated however, as possible interactions between these two inputs may exist^15^. The interpretation of our findings regarding the greater ventilatory response should thus be considered in relation to both feedforward and feedback mechanisms.

### BWS and ${\dot{\text{V}}}_{{\text{E}}}$–${\dot{\text{V}}}\text{CO}_{{2}}$ linkage during sinusoidal walking

As shown in Fig. 5, the *Amp* ratio in the ${\dot{\text{V}}}_{{\text{E}}}$ response was closely related to the *Amp* ratio in the ${\dot{\text{V}}}\text{CO}_{{2}}$ response. Note that the intercept of the regression lines for all three BWS conditions were close to zero, suggesting that the ${\dot{\text{V}}}_{{\text{E}}}$ did not change when there was no change in the metabolic demand (${\dot{\text{V}}}\text{CO}_{{2}}$). It has been recognized that (i) the ${\dot{\text{V}}}_{{\text{E}}}$ temporally follows the ${\dot{\text{V}}}\text{CO}_{{2}}$ irrespective of the type of work rate forcing^2,54,55^, and (ii) there would be minimal contribution to the ventilation changes from the neural signals through the motor activity such as neural feedforward and/or feedback inputs under sinusoidal walking^2,12,18,35^. Therefore, the underlying mechanism involved in the ventilatory response to sinusoidal workforce, showing a tight link between ${\dot{\text{V}}}_{{\text{E}}}$ and ${\dot{\text{V}}}\text{CO}_{{2}}$, could be explained by humoral factors (e.g., the metabolic rate) rather than the motor activity^2^.

In contrast, it is interesting that we observed marked differences in the slope of the regression line of the *Amp* ratio in ${\dot{\text{V}}}_{{\text{E}}}$ as a function of ${\dot{\text{V}}}\text{CO}_{{2}}$ across the three BWS conditions (50%BW: 1.34, 70%BW: 1.03, 100%BW: 0.91). It is conceivable that the observed greater ventilatory response under reduced BW condition was regulated by inputs other than metabolic inputs (i.e., neural inputs, including central command and muscle afferent feedback) at the given ${\dot{\text{V}}}\text{CO}_{{2}}$ value (at the equivalent metabolic rate). In other words, the neural drives via exercising muscles could be partly related to the steeper slope of the ${\dot{\text{V}}}_{{\text{E}}}$–${\dot{\text{V}}}\text{CO}_{{2}}$ linkage rather than the metabolic inputs^2,18,56,57^. Several studies have described possible mechanisms in which group III and IV muscle afferent feedback in humans contributes significantly to the ventilatory response during exercise^9,11^. The precise mechanism of the afferent feedback mediating the observed ventilatory response cannot be determined by our present experiments because the activity of muscle afferent fibers was not measured directly. However, the greater ventilatory response under reduced BW conditions may have at least partially occurred via muscle afferents in exercising muscles during sinusoidal walking, which may have been activated by the increasing step frequency. It must also be kept in mind this may be a result of an integration of muscle afferent activation and central command, however.

### BWS and lower-limb muscle activities during sinusoidal walking

The *Amp* ratio in the TA muscle increased as the BWS increased, whereas that in the GAS muscle decreased. A strong positive correlation between the *Amp* ratio in ${\dot{\text{V}}}_{{\text{E}}}$ and the TA muscle activity was also revealed (r = 0.99, p < 0.001), as was a strong negative correlation between the *Amp* ratio in ${\dot{\text{V}}}_{{\text{E}}}$ and the GAS muscle activity (r =  − 0.94, p < 0.001). These findings indicate that the relative TA muscle activity with increased amplitude may have contributed significantly to the greater ventilatory response under the BWS conditions.

It has been reported that the gastrocnemius muscle showed a strong load sensitivity and that its EMG activity decreased with body unloading^26^. Sensitivity of the gastrocnemius EMG activity to body unloading during locomotion has been observed in cats^58,59^ and humans^26,60^, and the input from the extensor load receptors, i.e., Golgi tendon organs, is thought to play a major role in the activation of ankle extensors during locomotion^58^. We thus speculate that the decrease in the *Mx* and the *Amp* values in the GAS muscle with the increase in BWS in the present study may be related to the decrease in the input from load receptors and its anti-gravity function.

In contrast, several research groups have reported that the TA muscle activity was unaffected by BWS^22,26,27^ and that a gravity-dependent function that can be seen in ankle extensors is absent for ankle dorsi-flexors^28^. Our present findings regarding the *Mx* of the TA muscle are consistent with these studies^22,26,27^, but the increase in the *Amp* response of the TA muscle is presumably related to the alteration in the locomotion parameters (the significant increase in step frequency) under BWS. We speculate that the TA muscle may have experienced significant activation during sinusoidal walking due to the significant increase in step frequency under BWS while the BWS attenuated the activity of extensor muscles, i.e., anti-gravity muscles. In an investigation of subjects’ muscle activity when walking at different cadences at the same speed, the TA muscle activity showed minimal change at the subjects’ preferred cadence and increased with faster or slower cadences^61^. This could be a part of motor control processes in which humans adapt their muscle activity for a specific walking frequency, which in turn optimizes the overall metabolic cost during walking^61^.

In addition, the investigation by Donelan and Kram of the effects of gravity on the stance and swing time at a given walking speed revealed that the stance time decreased significantly at lower gravity whereas the swing time was unaffected by a decrease in gravity^29^. This indicates that the swing time is not determined by a gravitational contribution^29^. Therefore, the relative TA muscle activity with increased amplitude under BWS appears to be related to the locomotion frequency (i.e., step frequency) but may not be related to changes in the duration of the swing phase.

Taken together, the past and present findings indicate that the TA muscle may have activated during sinusoidal walking due to the significant increase in step frequency under BWS, and the significant positive correlation that we observed between the *Amp* ratio in ${\dot{\text{V}}}_{{\text{E}}}$ and the TA muscle provides evidence that the relative TA muscle activity with increased amplitude under BWS contributed to the greater ventilatory outcomes (the *Amp* ratio in ${\dot{\text{V}}}_{{\text{E}}}$ and the ${\dot{\text{V}}}_{{\text{E}}}$–${\dot{\text{V}}}\text{CO}_{{2}}$ relationship) during sinusoidal walking. Based on indirect measures of neural activity (i.e., iEMG), it is conceivable that the greater relative TA muscle activity stimulated the ventilation, possibly via the muscle afferents within the TA muscle, although the precise individual contributions of neural factors (afferent feedback and central command) to the ventilatory response remain unknown.

### Study limitations

Several limitations may affect the interpretation of our data. First, the BWS apparatus that was also used in our previous studies could result in relatively large fluctuations of vertical force, and such fluctuations are not negligible^21,25^. Such fluctuations of vertical force presumably contributed to the alterations of locomotion parameters. Second, walking cadence is influenced mainly by walking speed rather than body-weight unloading^31^. However, our sinusoidal protocols of walking with BWS in which the treadmill speed was sinusoidally changed between 3 and 6 km h^−1^ may have induced the augmentation of the participants’ step frequency, and the interaction of BWS and sinusoidal changes in the walking speed may have induced the greater step frequency and the greater TA muscle activation.

Third, we did not assess the participants’ cardiometabolic characteristics (i.e., the ${\dot{\text{V}}}\text{O}_{{{\text{2max}}}}$ as well as the ventilatory threshold) prior to the experimental protocol administration. The maximal oxygen uptake values for the present participants can be estimated as approx. 2.86–3.13 L min^−1^ referring to previous papers^62–64^. Therefore, the observed ${\dot{\text{V}}}\text{O}_{{2}}$ values (which varied from 0.72 to 1.05 L min^−1^ in accord with sinusoidal workload) varied from 23 to 37% of the estimated ${\dot{\text{V}}}\text{O}_{{{\text{2max}}}}$ for our participants, which is substantially below the ventilatory threshold. We recruited healthy young males with no cardiorespiratory diseases, and thus their peak aerobic capacity may not be below the average range, i.e., the 50th percentile ${\dot{\text{V}}}\text{O}_{{{\text{2max}}}}$ of men aged 20–29 years^64^. However, without having measured ${\dot{\text{V}}}\text{O}_{{{\text{2max}}}}$ data, it is still uncertain how potential interaction effects between BWS and individual fitness levels may influence the ventilatory response.

Finally, only healthy young men were examined. While unlikely, based on previously cited evidence^12,13,20^, there is a remote possibility that currently undetermined sex differences could exist. We must acknowledge that the precise interaction effects of BWS and sex on the ventilatory response are still uncertain. Similarly, walking capability in particular under fluctuating walking speeds is a fundamental mode of transportation in our daily lives, and it remains unclear whether our present findings are generalizable across sex, and other confounding factors such as different age groups, cardiometabolic disease status, and/or training status. Further studies evaluating various populations would be valuable.

## Conclusions

Using a body weight support system under sinusoidal walking, we observed that the tibialis anterior muscle activity was unaffected by reduced body weight but responded with increased amplitude. The tibialis anterior muscle presumably experiences significant activation during the swing phase of sinusoidal walking due to the significant increase in step frequency under body weight support. We also detected a significant positive correlation between the amplitudes of sinusoidal variations in ventilation and the tibialis anterior muscle activity. The greater amplitude in the tibialis anterior muscle may have been a potent stimulus for the greater response of ventilation during sinusoidal walking, which may be involve at least in part the upward neural information from group III and IV afferents. However, we were unable to differentiate the involvements of peripheral afferent feedback from central command without direct measures. Our findings using body-weight support and sinusoidal exercise protocols have implications for understanding the link between the muscle activities and ventilation during human locomotion and for a clear evaluation of cardiorespiratory and muscle dynamics in response to exercise (Supplementary Information S1).

### Supplementary Information

Supplementary Information.

## Data Availability

The authors confirm that the data sets used in this study are available within the article and/or its supplementary information files.
